# Unlocking the Power of Late-Evening Snacks: Practical Ready-to-Prescribe Chart Menu for Patients with Cirrhosis

**DOI:** 10.3390/nu15153471

**Published:** 2023-08-05

**Authors:** Laura Leoni, Filippo Valoriani, Riccardo Barbieri, Martina Pambianco, Martina Vinciguerra, Chiara Sicuro, Antonio Colecchia, Renata Menozzi, Federico Ravaioli

**Affiliations:** 1 Division of Metabolic Diseases and Clinical Nutrition, Department of Specialistic Medicines, University Hospital of Modena and Reggio Emilia, Largo del Pozzo 71, 41125 Modena, Italy; leoni_laura@hotmail.it (L.L.); filippo.valoriani@aou.mo.it (F.V.); riccardo.barbieri@aou.mo.it (R.B.); martina.vinciguerra@aou.mo.it (M.V.); renata.menozzi@unimore.it (R.M.); 2 Gastroenterology Unit, Department of Medical Specialties, University Hospital of Modena, University of Modena & Reggio Emilia, 41121 Modena, Italy; pambiancomartina@gmail.com (M.P.); chiarasicuro44@gmail.com (C.S.); antonio.colecchia@unimore.it (A.C.); 3 Department of Medical and Surgical Sciences (DIMEC), University of Bologna, 40138 Bologna, Italy

**Keywords:** chronic liver disease, nutrition, ACLD, advanced liver diseases, hepatic encephalopathy, ascites, portal hypertension, cirrhosis, LES, supplementation, sarcopenia, muscle, fatigue

## Abstract

The efficacy of the late-evening snack (LES) has been extensively studied due to the impact of the longest intermeal duration occurring at night in patients with cirrhosis. While actual clinical guidelines on nutrition in chronic liver disease recommend an LES, no specific nutritional compositions have been reported by the European Association for the Study of the Liver (EASL) and the European Society for Clinical Nutrition and Metabolism (ESPEN). Late-evening snacks vary greatly among studies, including natural foods and/or nutritional supplements, yet oral supplements still need to fully meet the LES’s nutritional composition. In addition, many hepatologists need to gain experience in nutritional approaches and have access to registered dieticians who can help them manage patients with liver disease. Therefore, this review study aims to summarise evidence regarding using LESs and the mechanisms behind long starvation in patients with cirrhosis. It also provides a practical nutritional guide with several LES options based on common natural foods tailored to special patients’ nutritional requirements and geographical backgrounds. In preventing accelerated starvation and related protein malnutrition and sarcopenia in patients with cirrhosis, the nutritional composition of LESs is essential. The proper and straightforward application of the LES’s rational nutrition is an advantage to cirrhotic patients and should be carried out by healthcare professionals to enhance the overall liver function and nutritional status of patients with cirrhosis.

## 1. Introduction

A late-evening snack (LES) has been recommended as a strategy to address the nutritional needs of patients with liver diseases. Hepatologists often lack the resources, time, or expertise to adequately address these nutritional needs, leading to ineffective treatment prescriptions and consequences such as sarcopenia and hepatic encephalopathy. The optimal duration of LES administration is still being determined. However, the importance of shortening fasting intervals between meals, particularly during the longest intermeal duration at night, has been emphasised. An LES is a simple, safe, and cost-effective intervention to prevent catabolism and reverse sarcopenia. This review article aims to summarise the current evidence about the prescription of LESs in advanced chronic liver disease patients; moreover, we will offer a practical, nutritional, and graphical guide with several LES options based on common natural foods tailored to special patients’ nutritional requirements and their geographical backgrounds.

## 2. Data Sources and Searches

We searched English language publications on MEDLINE, Ovid, In-Process, Cochrane Library, EMBASE, and PubMed up to June 2023. Literature searches were performed using the following keywords: Late-evening snack, snack, nutritional management, cirrhosis, liver diseases, chronic liver disease, advanced chronic liver disease, chronic disease, cirrhosis, sarcopenia, sarcopenic, muscle depletion, muscle mass, hepatic encephalopathy, and ascites.

## 3. Impact of Malnutrition in Patients with Liver Cirrhosis

Cirrhosis, also known as advanced chronic liver disease (ACLD), is the end stage of progressive liver fibrosis, in which regenerative hepatic nodules replace the hepatic architecture as a consequence of chronic liver inflammation, and it eventually determines liver failure; cirrhosis is widely prevalent and is associated with high morbidity and mortality [1]. An initial asymptomatic phase characterises the disease, called compensated cirrhosis (cACLD), which evolves to a symptomatic stage (decompensated dACLD), defined as the first occurrence of liver events such as ascites, esophageal variceal bleeding (EVB), and hepatic encephalopathy (HE); these complications lead to frequent hospitalisation, infections, impaired quality of life, and high mortality [2]. The management of liver cirrhosis is focused on treating the causes and complications [1].

Malnutrition is a condition of altered body composition and cell functions secondary to insufficient protein and energy intake [3]. The diagnosis, assessment, and grading of malnutrition, according to the Global Leadership Initiative on Malnutrition (GLIM) [4] and the European Society for Clinical Nutrition and Metabolism (ESPEN) [5], requires the combination of a phenotypical criterion (weight loss, reduced body mass index or reduced muscle mass) and an etiological one (reduced food intake and assimilation and the presence of acute/chronic disease-related inflammation).

The prevalence of malnutrition in ACLD ranges from 5–92%, with high variability, which implies difficulties in malnutrition assessment and a knowledge gap [6]. Malnutrition prevalence increases with increasing disease severity (dACLD), but frequently malnutrition can be found even in patients in a compensated stage [7,8]. Malnutrition represents a complication of ACLD, being an independent negative predictor of disease progression and outcome (higher hospitalisation rate and mortality) [9]. Furthermore, malnutrition can be considered a predictor of other complications of ACLD [10], with a strong correlation with HE and infections [11,12].

Malnutrition in patients with cirrhosis shows a multifactorial etiology, which includes poor energy-protein intake, inflammation, malabsorption and intestinal protein loss, gut microbiome dysbiosis, nutrient metabolism dysregulation with decreased hepatic protein synthesis, hormonal disturbances, and hypermetabolism; moreover, external behaviours such as fasting periods and alcohol consumption can contribute to malnutrition [6]. In addition, other malnutrition-related diagnoses, such as sarcopenia and frailty, are commonly associated with liver cirrhosis [13,14,15]. Sarcopenia is defined as a progressive skeletal muscle depletion that involves a loss of muscle mass and strength function, and it is linked with increased adverse outcomes and mortality [16]. The prevalence of sarcopenia in cirrhotic patients ranges from 40 to 70%, with a large variability depending on the population evaluated (sex, ethnicity, degree of liver failure), methods of assessment, and the definition of sarcopenia used [17,18,19]. A meta-analysis of 22 published studies in the Journal of Hepatology in 2022 by X. Tantai et al. highlights that the overall prevalence of sarcopenia among patients with ACLD is 37.5%, with a higher prevalence in males, patients with alcohol-related liver disease, and patients with greater severity of cirrhosis (28.3% in Child-Pugh A, 37.9% in Child-Pugh B, and 46.7% in Child-Pugh C) [20]. The same authors show that sarcopenia is associated with an approximately two-fold higher risk of death in patients with cirrhosis and mortality rates at 1, 3, and 5 years of 23.4%, 35.7%, and 54.7%, respectively [20]. This high mortality risk is related to an increased risk of falls, fractures, reduced quality of life, and the development of liver-related complications [21]. Patients with liver disease are highly susceptible to sarcopenia due to multiple factors. These include symptoms like nausea, early satiety, and delayed gastric emptying caused by ascites and enteropathy. Anorexia due to elevated levels of leptin and tumour necrosis factor (TNF-a), physical inactivity, and obesity are also significant contributors. Additionally, hypermetabolism resulting from systemic inflammation, inadequate nutrition intake, and low socioeconomic status, commonly associated with alcoholism, also play a crucial role. Therefore, it is imperative to consider these factors while treating patients with liver disease to prevent or slow the onset of sarcopenia [22,23].

Recently, due to an increasing diagnosis of non-alcoholic fatty liver disease (NAFLD) related cirrhosis, more cirrhotic patients with overweight and obesity are observed [24]. Sarcopenic obesity is a term used to define the co-presence of muscle mass depletion and strength reduction with an excess of visceral adiposity; in this case, sarcopenia can be highly overlooked due to the presence of body weight excess [25]. Obesity and sarcopenic obesity worsen the prognosis of cirrhotic patients [26] and are associated with poor transplant waitlist mortality [27,28]. Given these observations, malnutrition and sarcopenia should be promptly recognised and treated. Current treatment strategies for sarcopenia in liver cirrhosis, which are mainly in investigational stages, include hormone replacement, antibiotics and gut microbiota manipulation, ammonia reduction, myostatin antagonists, nutritional supplementation such as branched-chain amino acids (BCAA) and L-carnitine supplements, and treatment of portal hypertension [29]. Although nutritional interventions and exercise, particularly resistance training, have been considered the main strategies to prevent sarcopenia in various populations [17,21,30].

## 4. Nutritional Requirements in Liver Cirrhosis Patient

The energy requirements of cACLD patients are not greater than those of healthy individuals (calculated as REE × 1.3), while their recommended protein intake is 1.2 g/kg/day; specific nutrition counselling should be provided to modify patients’ behaviour educating them about the benefits of a healthy diet adapted to their clinical condition. Nutrition counselling and diet prescriptions need to be shaped in response to the severity of the disease [31]. During the natural course of the disease, ACLD patients may exhibit a decreased appetite with a reduction in dietary intake, especially following the first hepatic decompensation event, exactly when energy expenditure is increased [32]. According to the last nutritional guidelines, a daily energy intake of at least 30–35 kcal/kg body weight in non-obese cirrhotic patients is recommended [31,33]. In patients with ascites and fluid retention, the energy intake should be calculated on dry weight, using post-paracentesis weight or subtracting a weight percentage depending on the fluid retention grade: mild, 5%; moderate, 10%; severe, 15%; additional 5% in those presenting bilateral pedal oedema to the knees [33]. In the case of body mass index (BMI) ≥ 30 kg/m^2^, weight-based energy intake recommendations may be modified to 25–35 kcal/kg/day for patients with BMI 30–40 kg/m^2^ and 20–25 kcal/kg/day for patients with BMI ≥ 40 kg/m [34], even if data are lacking. As for protein intake, the recommended daily protein intake is 1.2–1.5 g/kg ideal body weight [31,33], although ESPEN advises specifically to provide 1.5 g/kg/day in patients with malnutrition and muscle depletion [31]. For those with cACLD, it is advised to obtain their protein intake from a balanced combination of sources. This includes one-third of dairy protein (which contains casein), one-third of vegetable protein (which is rich in branched-chain amino acids), and one-third of animal protein (which is of high quality). Animal proteins are rich in aromatic amino acids not metabolised by skeletal muscle and may worsen HE if present. In the case of the ACLD patient who expresses a preference for different dietary habits, it is acceptable to make changes to prioritise their overall daily protein intake rather than focusing exclusively on exact proportions [35,36].

According to the established nutritional guidelines, it is advisable to divide one’s daily food intake into three primary meals, namely early breakfast, lunch, and dinner, while also incorporating three snacks, which may be consumed in the mid-morning, mid-afternoon, and late evening. Additionally, it is recommended to consume smaller, frequent meals, with no more than a 3–4 h gap between each meal, to maintain a consistent meal schedule and minimise prolonged fasting periods [21,31,33].

## 5. Rationale and Pathophysiological Principles of the Late-Evening Snack (LES)

Skeletal muscle mass results from a balance between muscle protein synthesis, which occurs during the fed state, and protein breakdown, which occurs during the fasting state. In patients with cirrhosis, this balance is characterised by reduced rates of body protein synthesis and increased rates of body protein breakdown during fasting periods due to abnormal metabolism [37,38]. In particular, cirrhosis shows a state of accelerated starvation after a short fasting period, with a change in the normal substrate usage, leading to an early shift from glucose to lipid and protein utilisation for energy due to a reduction in hepatic glycogen storage capacity, implying the need to generate glucose from alternate sources [39]. After overnight fasting, cirrhotic patients exhibit a different metabolic pattern than healthy individuals. While healthy individuals typically develop this pattern after 2–3 days of starvation, cirrhotic patients experience increased fat oxidation and gluconeogenesis while glucose oxidation and glycogenolysis decrease. Gluconeogenesis and protein oxidation lead to increased consumption of amino acids and skeletal muscle protein loss, which results in sarcopenia [40]. Furthermore, ACLD patients frequently experience complications like insulin resistance and hepatogenic diabetes. These conditions further intensify the process of muscle wasting.

**Figure 1:**
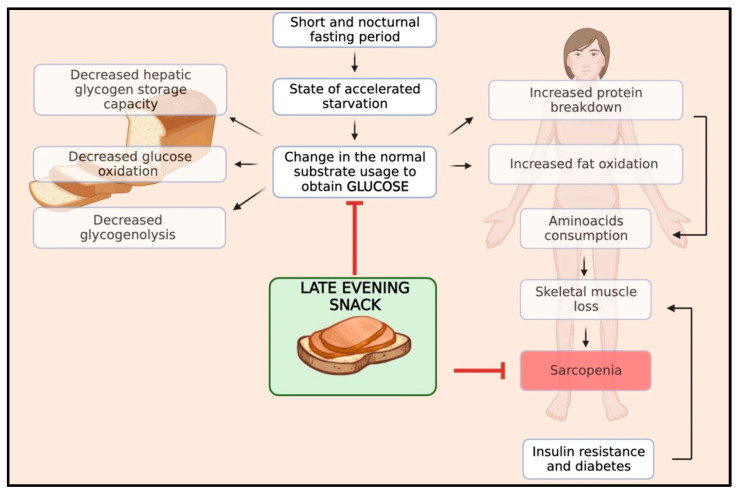
Impact of an LES on the pathophysiology of sarcopenia in cirrhosis.

Therefore, the optimal management of meal timing and frequency is of the utmost importance in preventing sarcopenia in patients with ACLD. Protein homeostasis, a balance between protein synthesis and breakdown, is disrupted in these patients, skewing toward elevated protein degradation, particularly during the protracted nocturnal fasting period. The interval from dinner to breakfast typically represents the longest fasting span, during which proteolysis is favoured over protein synthesis, thereby escalating muscle wasting [41]. By harnessing the potential benefits of nutritional interventions, such as late-evening snacks (LES), we can effectively curtail the duration of the night fasting period, thereby extending the patient’s fed state, facilitating the accelerated progression to a catabolic state and reversing sarcopenia. Moreover, an LES has been shown to stabilise blood glucose levels, curbing abrupt glucose and insulin spikes and promoting overall metabolic homeostasis. Therefore, an LES serves a dual purpose in ACLD patients by aiding in muscle preservation as well as managing typical cirrhotic implications such as insulin resistance complications. In Figure 1, we schematised the pathophysiology and rationale of an LES in patients with ACLD.

## 6. Evidence of an LES in Patients with ACLD

While the reasoning behind using an LES in patients with ACLD is understood, the concrete evidence pertaining to its benefit in disorder complications and overall prognosis remains partially unexplored, and its research seems to progress in a discontinuous or sporadic way. The concept of an LES for patients with cirrhosis was initially established by Swart et al. [42] in the late 1980s through a randomised controlled trial (RCT). This study, which observed a cohort of nine cirrhotic patients admitted to the ward for a few days, noted an improvement in nitrogen balance in patients who received evening meals. Subsequent studies, particularly from the Asia region, have produced a significant body of evidence supporting the clinical effectiveness of an LES in patients with ACLD. Indeed, a systematic review of trials about an LES in cirrhotic patients published up to December 2011 summarised the results of 15 articles, six short-term studies that analysed substrate utilisation and nine longer-term studies that examined the impact of an LES on clinical nutritional indices. Results showed that an LES reverses the substrate utilisation pattern in cirrhosis, switching to a more physiological pattern of glucose utilisation instead of lipid oxidation and gluconeogenesis. Moreover, an LES led to an increase in the respiratory quotient, an improvement of nitrogen balance, independently of the composition or formulation used, improvement of fat-free mass and primarily skeletal muscle mass (especially in subjects with less advanced disease). Additional benefits may include a reduction in the frequency and severity of HE because skeletal muscle mass preservation may enhance non-hepatic ammonia removal since skeletal muscle is the main ammonia disposal organ via glutamine synthesis [41]. More recently, new studies have been published evaluating changes in biochemical and energy parameters before and after an LES intervention. A systematic review and meta-analysis by Ying-jje Guo et al., published in 2018, combined 14 clinical studies from 1997 to 2017 with a population of 478 patients who received an LES for at least one week. Results showed that the levels of serum albumin, prealbumin, and cholinesterase, which are hepatic synthetic metabolism biomarkers, were significantly increased with the LES treatment [43]. Moreover, a recent paper by Hiraoka et al. [44] has shown, in a cohort of 33 Japanese liver cirrhotic patients, that a lifestyle intervention based on the supplementation of BCAA, and walking exercise improves muscle volume and strength, suggesting a possible improvement in sarcopenia. Finally, a recent meta-analysis and systematic review by Chen-Ju Chen et al. found that an LES administration reduced ALT, AST, PT, and ammonia levels in patients with liver diseases and reduced ascites and HE occurrence. In the studies considered, it emerged that long-term administration is preferable to short-term treatment; however, there has yet to be a consensus on the length of LES administration required to improve liver function [45]. Otherwise, the effect of an LES on other complications of cirrhosis, such as the real impact on sarcopenia prevention and decreased mortality or need for transplantation, is yet unknown. In Table 1, we summarised the current evidence in the application of an LES in patients with ACLD.

## 7. Nutritional Composition of the Late-Evening Snack (LES)

To mitigate accelerated starvation and related proteolysis, a key dietary strategy involves consuming food every 4 to 6 h, thereby reducing the fasting intervals between meals. While the longest intermeal duration typically occurs at night, the efficacy of a late-evening snack (LES) has been extensively researched. The Clinical Guidelines on Nutrition in chronic liver disease by the European Association for the Study of the Liver [33] and the European Society for Clinical Nutrition and Metabolism (ESPEN) [31] recommend an LES but do not specify the nutritional composition. Recent reviews and meta-analyses, however, indicate that an LES containing complex carbohydrates and proteins can reduce lipid oxidation and improve nutritional status, nitrogen balance, muscle mass, liver function, and overall quality of life in patients with cirrhosis [41,43,45]. The International Society for Hepatic Encephalopathy and Nitrogen Metabolism 2013 consensus article [34] suggests that an LES should contain at least 50 g of complex carbohydrates. In most clinical studies, various night meal compositions seem effective as they contain a reasonable amount of complex carbohydrates (at least 30 g/LES) and protein (about 13.5 g/LES) [43,45,69]. The energy intake from an LES may vary approximately between 200 and 250 kcal [43,45,69]. However, no specific recommendations are available for other nutrients. Due to the pathophysiology of ascites, a moderate dietary sodium intake (60 mmol/day = 1360 mg/day) is typically advised.

When recommending a low-sodium diet, it is important to consider its poor palatability, which may lead to reduced energy and protein intake. Its potential benefit in treating ascites should be balanced against the increased risk of even lower food consumption [31]. Studies of the LES have shown varying compositions, including natural foods and nutritional supplements with varying BCAA content. However, oral food supplements can only partially satisfy the recommended nutrition for SLE, in particular, due to an inappropriate glucoside intake from a qualitative–quantitative point of view. Therefore, from published evidence, we reported here in Table 2 the summary of the nutritional composition of the LES proposed for patients with ACLD.

## 8. Practical Approach to a Late-Evening Snack (LES) for ACLD

The current literature on what should be included in a late-evening snack (LES) for patients diagnosed with liver diseases is quite varied, thus necessitating the need for a more precise consensus. Hepatologists possess limited knowledge regarding the exact composition and prescription of an LES, and there is a scarcity of dieticians specialising in patients with liver disease. Our objective is to present practical and easy-to-prescribe examples of LESs for ACLD patients, supported by the most recent evidence. We have proposed 43 different options that use commonly available natural foods to achieve the nutritional characteristics and objectives outlined in Table 2. These LES options were developed using validated food composition databases [70,71,72].

**Figure 2:**
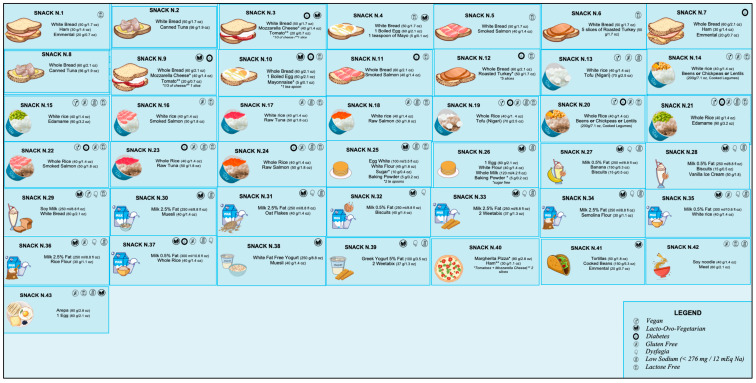
Proposal for late-evening snacks. For bromatologic compositions, see Supplementary File.

Figure 2 reported all the LESs developed (Supplementary File shows the specific composition for each snack). In order to ensure that individuals diagnosed with ACLD receive the appropriate treatment for their clinical condition or personal preferences, specific symbols are utilised to indicate the suitability of a particular LES. For example, a gluten-free LES is offered for patients with celiac disease, while a low-sugar LES is recommended for those with diabetes. A modified texture LES is available for individuals with dysphagia, whereas a lactose-free LES is provided for those with lactose intolerance. Additionally, a low sodium (<276 mg/snack) LES is recommended for patients with severe ascites. Furthermore, we have identified alternative oral nutritional supplements that could potentially serve as an LES based on their compositions (Table 3).

**Figure 3:**
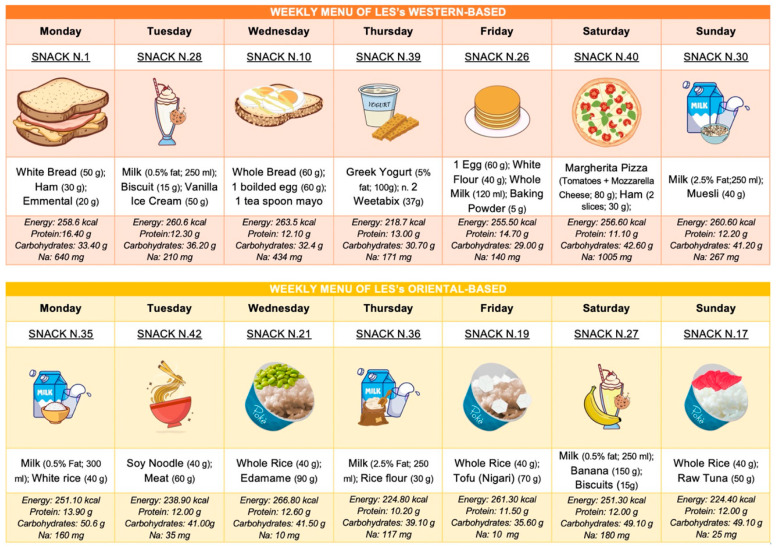
Weekly meal plans for LESs based on different geographic cultures.

Finally, we also developed the LES by considering gastronomic cultures worldwide and specific dietary patterns such as vegan and lacto-ovo vegetarian. As shown in Figure 3, we have provided two weekly meal plans for the LES—one inspired by a typical Western diet and the other by an Oriental diet.

## 9. Conclusions

While the potential of the LES intervention in managing the issues and its positive influence on ACLD patients’ quality of life is evident from extant research, further investigation is required to delineate its direct impact on survival outcomes. Nonetheless, it underscores the importance of nutritional adjustments in managing ACLD, making an LES an advised practice within the broader framework of these patients’ nutritional management strategy. However, it is critical to individualise dietary strategies accounting for the patient’s overall health conditions, preferences, and cirrhosis-specific complications to maximise the intervention’s efficacy. The LES supplementation, ideally rich in complex carbohydrates and with a moderate amount of protein, influences physiological regulation by providing a consistent supply of amino acids for protein synthesis. This method reduces the onset of proteolytic catabolism to sustain glycogen levels, ultimately diminishing protein and fat degradation, which is essential in maintaining muscle mass and improving the patient’s nutritional status.

## Figures and Tables

**Table 1:** Summary of the evidence published on an LES in patients with liver cirrhosis.

Authors	Year	Region	n. Cases	n. Controls	Study Type	Major Findings
Swart et al. [42]	1989	NL	9	.	RCT (unblinded)	LES improves efficiency of nitrogen balance in LC
Zillikens et al. [46]	1993	NL	8	8	Crossover	LES improves nitrogen balance during the night in LC but not in HE
Verboeket-van De Venne et al. [47]	1995	NL	8	23	Crossover	LES reduces episodes of protein catabolism during the day in LC
Chang et al. [48]	1997	China	16	8	Crossover	LES effectively diminishes fat and protein oxidation in LC
Miwa et al. [49]	2000	Japan	12	14	Crossover	LES corrects abnormal metabolism and prevents malnutrition in LC.
Yamauchi et al. [50]	2001	Japan	14	.	Crossover	LES improves protein catabolism and lipolysis in LC.
Nakaya et al. [51]	2002	Japan	7	5	Crossover	LES based on BCAA mixture or carbohydrate-rich snacks can reduce fat oxidation in LC
Fukushima et al. [52]	2003	Japan	12	12	RCT	LES by BCAA administration improved serum albumin levels in LC more than daytime BCAA administration
Sako et al. [53]	2003	Japan	8	.	Crossover	LES with BCAA supplements provides relief from muscle cramping,
Sakaida et al. [54]	2004	Japan	11	.	Crossover	LES improves glucose intolerance in hospitalized LC.
Yamanaka-Okumura et al. [55]	2006	Japan	21	26	Crossover	LES with a 200-kcal rice ball improves the nutritional metabolism LC
Gheorghe et al.[56]	2005	Romania	122	.	.	LES improves HE
Nakaya et al. [57]	2007	Japan	19	19	RCT	Long-term oral supplementation with BCAA mixture as LES improves albumin levels and energy metabolism in LC
Plank et al. [56]	2008	NZ	51	52	RCT	LES leads to significant protein improvement, equivalent to 2 kg of lean tissue over 12 months.
Takeshita et al. [58]	2009	Japan	28	28	RCT	LES with BCAA supplement prevents liver function failure after TACE in LC patients with HCC
Ichikawa et al. [59]	2010	Japan	12	9	RCT (unblinded)	LES with BCAA improves sleep quality for LC without HE
Harima et al. [60]	2010	Japan	13	10	RCT	LES with BCAA-enriched nutrients may improve energy metabolism and glucose tolerance in LC with HCC
Yamanaka-Okumura et al. [61]	2010	Japan	15	24	RCT (unblinded)	LES may help maintain higher HRQOL in LC.
Kuroda et al. [62]	2010	Japan	20	15	RCT	LES with BCAA-enriched nutrients after RFA therapy for HCC improves nutritional status and quality of life of LC for 1-years
Sorrentino et al. [63]	2012	Italy	40	40	RCT	LES reduces the rate of refractory ascites in LC
Morihara et al. [64]	2012	Japan	10	10	RCT	LES with BCAA supplementation improves liver functioning and cognitive performance in LC patients who have undergone RFA for HCC.
Hidaka et al. [65]	2013	Japan	16	21	RCT	LES with BCAA administration reduced leg cramps in LC but did not improve quality of life.
Nojiri et al. [66]	2017	Japan	25	26	RCT	LES with EN formula (Aminoleban) improves HE in LC after RFA and reduce the risks for HCC recurrence.
Hiraoka et al. [44]	2017	Japan	33	.	Crossover	LES with BCAA and walking exercise improves muscle volume and strength in LC
Hou et al. [67]	2021	China	43	43	RCT	LES with carbohydrate-predominant significantly increases BCAA and decreases ammonia and glutamine after 6 months of supplementation.
Zhao et al. [68]	2023	China	63	28	RCT	LES increases the respiratory quotient in alcoholic LC. LES and BCAA were more effective than LES alone in improving serum isoleucine and the Fischer ratio.

Abbreviations: BCAA, branched-chain amino acid; EN, enteral nutrition; HCC, Hepatocellular carcinoma; HE, hepatic encephalopathy; HRQOL, Health-related quality of Life; LC, liver cirrhosis; LES, late-evening snack; NL, Netherland; NZ, New Zeland; RCT, randomised controlled trial; RFA, Radiofrequency ablation; TACE, transarterial chemo-embolisation.

**Table 2:** Suggested nutritional composition of the LES in patients with cirrhosis.

Energy (kcal)	Proteins (g)	Complex Carbohydrates (g)	Sodium (mg)
246 (200–275)	15 (11.5–18)	40 (25–55)	550 (10–1085)

**Table 3:** Compatible and commercial oral nutritional supplements such as an LES.

Brand	Name	Size (cc)	Energy (kcal)	Protein (g)	Fat (g)	Total CHOT(g)	MonoCHOT	DiCHOT	OligoCHOT	PoliCHOT
Nutricia	Cubitan©	200	256	18.00	7.00	28.40	X	X	X	
Nutricia	Diasip©	200	208	9.80	7.60	23.40	X	X	X	X
Nutricia	Forticare©	125	200	11.00	6.60	23.90	X	X	X	
Nutricia	Fortimel/12 measuring spoon©	69	300	15.00	10.50	30.30	X	X	X	
Nutricia	Fortimel Compact Protein©	125	300	18.00	11.80	30.50	X	X	X	
Nutricia	Nutilis Complete Stage 1©	125	300	12.00	11.70	36.40	X	X		
Nutricia	Nutridrink Compact©	125	300	12.00	11.70	37.10	X	X		
Abbott	Ensure Compact©	125	300	12.30	11.70	35.90	X	X		
Abbott	Ensure Compact Protein HMB©	125	300	18.00	12.50	28.00	X	X		
Abbott	Ensure Plus Drink©	200	300	12.50	9.80	40.40	X	X		
Abbott	Glucerna SR©	220	205	9.40	7.70	23.90	X	X		
Abbott	Prosure©	220	280	14.60	5.60	40.30	X	X		
Nestlè	Resource Repair©	200	254	18.00	6.00	32.00	X	X	X	
Nestlè	Resource Ultra©	125	281	17.50	11.00	28.00	X	X	X	
Nestlè	Resource Energy©	200	302	11.20	10.00	42.00	X	X	X	
Nestlè	Resource Drink©	200	250	18.80	7.00	28.00	X	X		
Nestlè	Meritene Crème©	125	212	12.00	9.10	20.00	X	X	X	
Fresenius Kabi	Fresubin Protein Energy Drink©	200	300	20.00	13.40	24.80	X	X	X	

Abbreviations: CHOT: Carbohydrates; MonoCHOT: Monosaccharides; DiCHOT: Disaccharides; OligoCHOT: Oligosaccharides; PoliCHOT: Polysaccharide.

## Data Availability

Not applicable.

## Supplementary Materials

The supporting information can be downloaded at: https://www.mdpi.com/article/10.3390/nu15153471/s1.
