# Supplementary material for: Unlocking the Power of Late-Evening Snacks: Practical Ready-to-Prescribe Chart Menu for Patients with Cirrhosis
Source: Nutrients. 2023 Aug 5;15(15):3471. doi: 10.3390/nu15153471 (PMC10420913; doi:10.3390/nu15153471)
Supplement: Supplementary file 1 [file nutrients-15-03471-s001.zip › nutrients-2529185-supplementary.pdf]

SANDWICHES

SNACK N.1

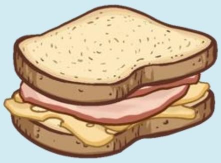

White Bread (50 g/1.7 oz)
Ham (30 g/1.4 oz)
Emmental (20 g/0.7 oz)

	g	Kcal	%
Protein	**16.40**	65.60	25.40
Fat	7.50	67.90	26.20
Carbohydrates	**33.40**	125.10	48.40
Total Energy		**258.60**	100.00
Sodium (mg)	640		

SNACK N.2

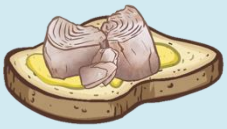

White Bread (50 g/1.7 oz)
Canned Tuna (56 g/1.9 oz)

	g	Kcal	%
Protein	**17.90**	71.60	29.30
Fat	5.70	50.90	20.90
Carbohydrates	**32.40**	121.30	49.80
Total Energy		**243.80**	100.00
Sodium (mg)	510		

SNACK N.3

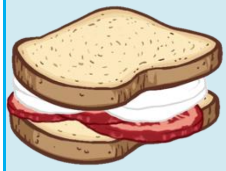

White Bread (50 g/1.7 oz)
Mozzarella Cheese* (40 g/1.4 oz)
Tomato** (20 g/0.7 oz)
1/3 of cheese /1 slice*

	g	Kcal	%
Protein	**11.80**	47.10	19.40
Fat	7.90	71.20	29.40
Carbohydrates	**33.20**	124.50	51.20
Total Energy		**242.60**	100.00
Sodium (mg)	413		

SNACK N.4

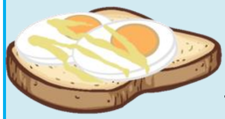

White Bread (50 g/1.7 oz)
1 Boiled Egg (60 g/2.1 oz)
1 teaspoon of Mayo (5 g/0.1 oz)

	g	Kcal	%
Protein	**11.70**	46.80	18.90
Fat	8.80	79.40	32.00
Carbohydrates	**32.50**	121.70	49.10
Total Energy		**247.90**	100.00
Sodium (mg)	1085		

SNACK N.5

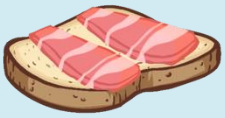

White Bread (50 g/1.7 oz)
Smoked Salmon (40 g/1.4 oz)

	g	Kcal	%
Protein	**14.40**	57.70	25.30
Fat	5.40	48.60	21.30
Carbohydrates	**32.50**	121.70	53.40
Total Energy		**228.00**	100.00
Sodium (mg)	1085		

SNACK N.6

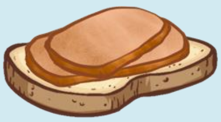

White Bread (50 g/1.7 oz)
5 slices of Roasted Turkey (50 g/1.7 oz)

	g	Kcal	%
Protein	**13.50**	54.00	24.30
Fat	4.40	39.50	17.80
Carbohydrates	**34.30**	128.50	57.90
Total Energy		**222.00**	100.00
Sodium (mg)	730		

SNACK N.7

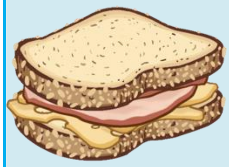

Whole Bread (60 g/2.1 oz)
Ham (30 g/1.4 oz)
Emmental (20 g/0.7 oz)

	g	Kcal	%
Protein	**16.80**	67.20	24.50
Fat	8.20	73.80	26.90
Carbohydrates	**33.30**	133.20	48.60
Total Energy		**274.20**	100.00
Sodium (mg)	640		

SNACK N.8

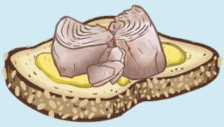

Whole Bread (60 g/2.1 oz)
Canned Tuna (56 g/1.9 oz)

	g	Kcal	%
Protein	**18.30**	73.20	28.10
Fat	6.40	57.60	22.10
Carbohydrates	**32.30**	129.20	49.80
Total Energy		**260.00**	100.00
Sodium (mg)	506		

SNACK N.9

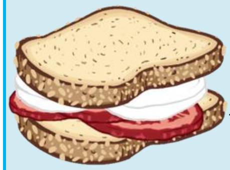

Whole Bread (60 g/2.1 oz)
Mozzarella Cheese* (40 g/1.4 oz)
Tomato** (20 g/0.7 oz)
1/3 of cheese/ 1 slice*

	g	Kcal	%
Protein	**12.20**	48.8	18.90
Fat	8.60	77.40	29.90
Carbohydrates	**33.10**	132.4	51.20
Total Energy		**258.6**	100
Sodium (mg)	410		

SNACK N.10

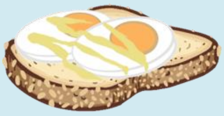

Whole Bread (60 g/2.1 oz)
1 Boiled Egg (60 g/2.1 oz)
Mayonnaise* (5 g/0.1 oz)
**1 tea spoon*

	g	Kcal	%
Protein	**12.10**	48.40	18.40
Fat	9.50	85.50	32.40
Carbohydrates	**32.40**	129.60	49.20
Total Energy		**263.50**	100.00
Sodium (mg)	434		

SNACK N.11

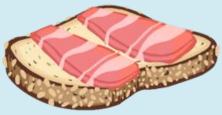

Whole Bread (60 g/2.1 oz)
Smoked Salmon (40 g/1.4 oz)

	g	Kcal	%
Protein	**14.80**	59.20	24.30
Fat	6.10	54.90	22.50
Carbohydrates	**32.40**	129.60	53.20
Total Energy		**243.70**	100.00
Sodium (mg)	1082		

SNACK N.12

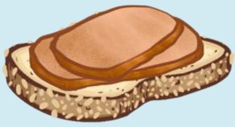

Whole Bread (60 g/2.1 oz)
Roasted Turkey* (50 g/1.7 oz)
**5 slices*

	g	Kcal	%
Protein	**13.90**	55.60	23.20
Fat	5.20	46.80	19.50
Carbohydrates	**34.20**	136.80	57.30
Total Energy		**239.20**	100.00
Sodium (mg)	730		

RICE POKE'

SNACK N.13

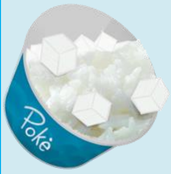

White rice (40 g/1.4 oz)
Tofu (Nigari) (70 g/2.5 oz)

	g	Kcal	%
Protein	**11.60**	46.50	18.30
Fat	7.20	65.20	25.60
Carbohydrates	**38.10**	142.90	56.10
Total Energy		**254.60**	100.00
Sodium (mg)	10		

SNACK N.14

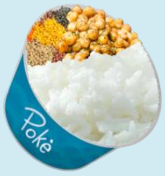

White rice (40 g/1.4 oz)
Beens **or** Chickpeas **or** Lentils
(200g/7.1 oz, Cooked Legumes)

	g	Kcal	%
Protein	**11.70**	46.90	18.40
Fat	1.80	15.90	6.30
Carbohydrates	**51.20**	191.90	75.30
Total Energy		**254.70**	100.00
Sodium (mg)	695		

SNACK N.15

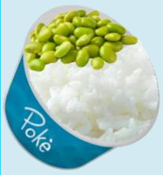

White rice (40 g/1.4 oz)
Edamame (90 g/3.2 oz)

	g	Kcal	%
Protein	**12.70**	50.80	19.60
Fat	4.70	42.70	16.50
Carbohydrates	**44.00**	165.10	63.90
Total Energy		**258.60**	100.00
Sodium (mg)	10		

SNACK N.16

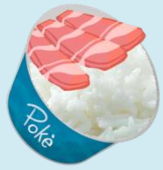

White rice (40 g/1.4 oz)
Smoked Salmon (50 g/1.8 oz)

	g	Kcal	%
Protein	**15.50**	62.00	28.70
Fat	2.50	22.40	10.40
Carbohydrates	**35.00**	131.40	60.90
Total Energy		**215.80**	100.00
Sodium (mg)	940		

SNACK N.17

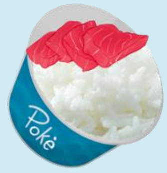

White rice (40 g/1.4 oz)
Raw Tuna (50 g/1.8 oz)

	g	Kcal	%
Protein	**13.60**	54.20	24.20
Fat	4.30	38.60	17.20
Carbohydrates	**35.10**	131.60	58.60
Total Energy		**224.40**	100.00
Sodium (mg)	25		

SNACK N.18

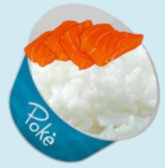

White rice (40 g/1.4 oz)
Raw Salmon (50 g/1.8 oz)

	g	Kcal	%
Protein	**12.00**	48.00	20.20
Fat	6.20	56.20	23.70
Carbohydrates	**35.50**	133.30	56.10
Total Energy		**237.50**	100.00
Sodium (mg)	52		

SNACK N.19

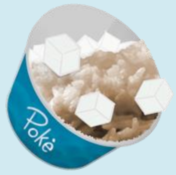

Whole Rice (40 g/1. 4 oz)
Tofu (Nigari) (70 g/2.5 oz)

	g	Kcal	%
Protein	**11.50**	46.00	17.70
Fat	8.10	72.90	27.80
Carbohydrates	**35.60**	142.40	54.50
Total Energy		**261.30**	100.00
Sodium (mg)	10		

SNACK N.20

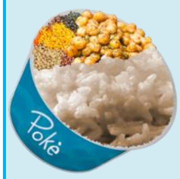

Whole Rice (40 g/1.4 oz)
Beens **or** Chickpeas **or** Lentils
(200g/7.1 oz, Cooked Legumes)

	g	Kcal	%
Protein	**11.60**	46.40	17.10
Fat	3.50	31.50	11.50
Carbohydrates	**48.70**	194.80	71.40
Total Energy		**272.70**	100.00
Sodium (mg)	690		

SNACK N.21

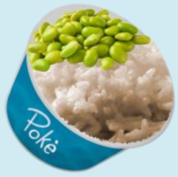

Whole Rice (40 g/1.4 oz)
Edamame (90 g/3.2 oz)

	g	Kcal	%
Protein	**12.60**	50.40	18.90
Fat	5.60	50.40	18.90
Carbohydrates	**41.50**	166.00	62.20
Total Energy		**266.80**	100.00
Sodium (mg)	10		

SNACK N.22

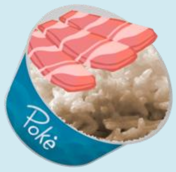

Whole Rice (40 g/1.4 oz)
Smoked Salmon (50 g/1.8 oz)

	g	Kcal	%
Protein	**15.40**	61.60	27.70
Fat	3.40	30.60	13.80
Carbohydrates	**32.50**	130.00	58.50
Total Energy		**222.20**	100.00
Sodium (mg)	940		

SNACK N.23

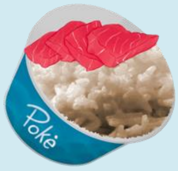

Whole Rice (40 g/1.4 oz)
Raw Tuna (50 g/1.8 oz)

	g	Kcal	%
Protein	**13.50**	54.00	23.40
Fat	5.20	46.80	20.20
Carbohydrates	**32.60**	130.40	56.40
Total Energy		**231.20**	100.00
Sodium (mg)	23		

SNACK N.24

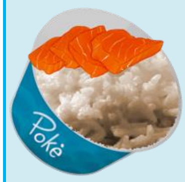

Whole Rice (40 g/1.4 oz)
Raw Salmon (50 g/1.8 oz)

	g	Kcal	%
Protein	**11.90**	47.60	19.60
Fat	7.10	63.90	26.20
Carbohydrates	**33.00**	132.00	54.20
Total Energy		**243.50**	100.00
Sodium (mg)	50		

PANCAKES

SNACK N.25

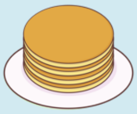

Egg White (100 ml/3.5 fl oz)
White Flour (45 g/1.6 oz)
Sugar* (10 g/0.4 oz)
Baking Powder (5 g/0.2 oz)
**2 te spoons*

	g	Kcal	%
Protein	15.70	62.60	26.70
Fat	0.30	2.80	1.20
Carbohydrates	45.20	169.60	72.10
Total Energy		**235.00**	100.00
Sodium (mg)	170		

SNACK N.26

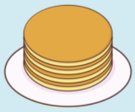

1 Egg (60 g/2.1 oz)
White Flour (40 g/1.4 oz)
Whole Milk (120 ml/4.2 fl oz)
Baking Powder * (5 g/0.2 oz)
**sugar free*

	g	Kcal	%
Protein	**14.70**	58.80	23.00
Fat	9.80	87.80	34.40
Carbohydrates	**29.00**	108.90	42.60
Total Energy		**255.50**	100.00
Sodium (mg)	140		

MILKSHAKES

SNACK N.27

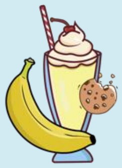

Milk 0.5% Fat (250 ml/8.8 fl oz)
Banana (150 g/5.3 oz)
Biscuits (15 g/0.5 oz)

	g	Kcal	%
Protein	**12.00**	48.20	19.20
Fat	2.10	19.20	7.60
Carbohydrates	**49.10**	193.90	73.20
Total Energy		**251.30**	100.00
Sodium (mg)	180		

SNACK N.28

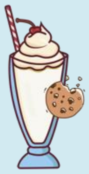

Milk 0.5% Fat (250 ml/8.8 fl oz)
Biscuits (15 g/0.5 oz)
Vanilla Ice Cream (50 g/1.8)

	g	Kcal	%
Protein	**12.30**	49.40	18.90
Fat	8.50	76.80	29.30
Carbohydrates	**36.20**	135.60	51.80
Total Energy		**260.60**	100.00
Sodium (mg)	210		

SNACK N.29

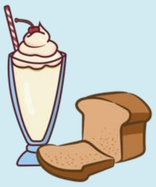

Soy Milk (250 ml/8.8 fl oz)
White Bread (60 g/2.1 oz)

	g	Kcal	%
Protein	**11.90**	47.40	17.80
Fat	5.00	45.20	16.90
Carbohydrates	**46.30**	173.70	65.30
Total Energy		**266.30**	100.00
Sodium (mg)	480		

MILK BASED

SNACK N.30

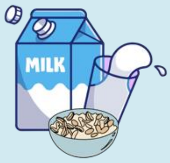

Milk 2.5% Fat (250 ml/8.8 fl oz)
Muesli (40 g/1.4 oz)

	g	Kcal	%
Protein	**12.20**	48.90	18.80
Fat	6.40	57.20	21.90
Carbohydrates	**41.20**	154.50	59.30
Total Energy		**260.60**	100.00
Sodium (mg)	267		

SNACK N.31

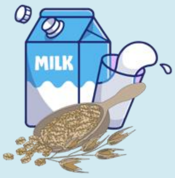

Milk 2.5% Fat (250 ml/8.8 fl oz)
Oat Flakes (40 g/1.4 oz)

	g	Kcal	%
Protein	**12.20**	48.90	20.30
Fat	3.50	31.50	13.10
Carbohydrates	**42.70**	159.90	66.60
Total Energy		**240.30**	100.00
Sodium (mg)	130		

SNACK N.32

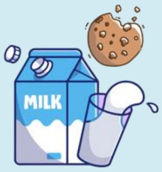

Milk 0.5% Fat (250 ml/8.8 fl oz)
Biscuits (40 g/1.4 oz)

	g	Kcal	%
Protein	**11.60**	46.60	18.10
Fat	3.70	32.90	12.80
Carbohydrates	**47.40**	177.80	69.10
Total Energy		**257.30**	100.00
Sodium (mg)	294		

SNACK N.33

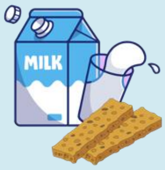

Milk 2.5% Fat (250 ml/8.8 fl oz)
2 Weetabix (37 g/1.3 oz)

	g	Kcal	%
Protein	**12.70**	50.80	21.00
Fat	4.50	40.40	16.70
Carbohydrates	**40.20**	150.80	62.30
Total Energy		**242.00**	100.00
Sodium (mg)	215		

SNACK N.34

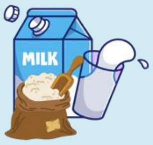

Milk 2.5% Fat (250 ml/8.8 fl oz)
Semolina Flour (30 g/1.1 oz)

	g	Kcal	%
Protein	**11.50**	45.80	20.90
Fat	4.20	37.30	17.20
Carbohydrates	**36.00**	135.10	61.90
Total Energy		**218.20**	100.00
Sodium (mg)	119		

SNACK N.35

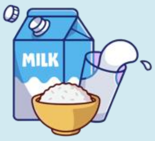

Milk 0.5% Fat (300 ml/10.6 fl oz)
White rice (40 g/1.4 oz)

	g	Kcal	%
Protein	**13.90**	53.60	22.00
Fat	0.80	7.60	3.00
Carbohydrates	**50.60**	189.90	75.00
Total Energy		**251.10**	100.00
Sodium (mg)	160		

SNACK N.36

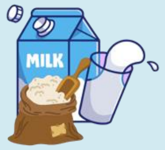

Milk 2.5% Fat (250 ml/8.8 fl oz)
Rice Flour (30 g/1.1 oz)

	g	Kcal	%
Protein	**10.20**	40.80	18.10
Fat	4.20	37.40	16.60
Carbohydrates	**39.10**	146.60	65.30
Total Energy		**224.80**	100.00
Sodium (mg)	117		

SNACK N.37

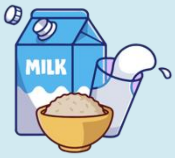

Milk 0.5% Fat (300 ml/10.6 fl oz)
Whole Rice (40 g/1.4 oz)

	g	Kcal	%
Protein	**13.80**	55.20	21.00
Fat	1.70	15.30	5.80
Carbohydrates	**48.10**	192.40	73.20
Total Energy		**262.90**	100.00
Sodium (mg)	150		

YOGURT BASED

SNACK N.38

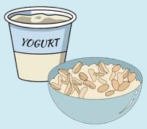

White Fat Free Yogurt (250 g/8.8 oz)
Muesli (40 g/1.4 oz)

	g	Kcal	%
Protein	**12.50**	49.90	21.20
Fat	4.60	41.50	17.60
Carbohydrates	**38.40**	144.20	61.20
Total Energy		**235.60**	100.00
Sodium (mg)	297		

SNACK N.39

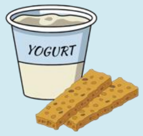

Greek Yogurt 5% Fat (100 g/3.5 oz)
2 Weetabix (37 g/1.3 oz)

	g	Kcal	%
Protein	**13.00**	51.80	23.70
Fat	5.70	51.70	23.60
Carbohydrates	**30.70**	115.20	52.70
Total Energy		**218.70**	100.00
Sodium (mg)	171		

OTHERS

SNACK N.40

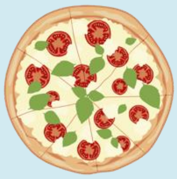

Margherita Pizza* (80 g/2.8 oz)
Ham** (30 g/1.1 oz)
**Tomatoes + Mozzarella Cheese*
*** 2 slices*

	g	Kcal	%
Protein	**11.10**	44.60	17.40
Fat	5.80	52.20	20.30
Carbohydrates	**42.60**	159.80	62.30
Total Energy		**256.60**	100.00
Sodium (mg)	1005		

SNACK N.41

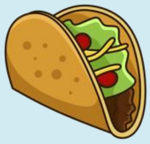

Tortillas (50 g/1.8 oz)
Cooked Beans (150 g/5.3 oz)
Emmental (20 g/0.7 oz)

	g	Kcal	%
Protein	**14.70**	58.80	23.10
Fat	8.30	74.70	29.20
Carbohydrates	**32.50**	121.80	47.70
Total Energy		**255.30**	100.00
Sodium (mg)	570		

SNACK N.42

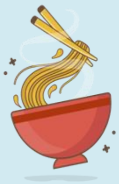

Soy noodle (40 g/1.4 oz)
Meat (60 g/2.1 oz)

	g	Kcal	%
Protein	**12.00**	47.90	20.10
Fat	4.10	37.20	15.60
Carbohydrates	**41.00**	153.80	64.30
Total Energy		**238.90**	100.00
Sodium (mg)	35		

SNACK N.43

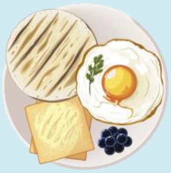

Arepa (80 g/2.8 oz)
1 Egg (60 g/2.1 oz)

	g	Kcal	%
Protein	**11.40**	45.80	19.10
Fat	9.20	82.90	34.60
Carbohydrates	**29.60**	111.00	46.30
Total Energy		**239.70**	100.00
Sodium (mg)	130		

LEGEND:

Vegan

Lacto-Ovo-Vegetarian

Diabetes

Gluten Free

Dysphagia

Low Sodium (< 276 mg / 12 mEq Na)

Lactose Free
